# Genome analyses provide insights into the evolution and adaptation of the eukaryotic Picophytoplankton *Mychonastes homosphaera*

**DOI:** 10.1186/s12864-020-06891-6

**Published:** 2020-07-11

**Authors:** Changqing Liu, Xiaoli Shi, Fan Wu, Mingdong Ren, Guang Gao, Qinglong Wu

**Affiliations:** 1 State Key Laboratory of Lake Science and Environment, Nanjing Institute of Geography and Limnology, Chinese Academy of Sciences, Nanjing, 210008 China; 2 University of Chinese Academy of Sciences, Beijing, 100049 China

**Keywords:** Picophytoplankton, *Mychonastes*, Genome, Adaptation

## Abstract

**Background:**

Picophytoplankton are abundant and can contribute greatly to primary production in eutrophic lakes. *Mychonastes* species are among the common eukaryotic picophytoplankton in eutrophic lakes. We used third-generation sequencing technology to sequence the whole genome of *Mychonastes homosphaera* isolated from Lake Chaohu, a eutrophic freshwater lake in China.

**Result:**

The 24.23 Mbp nuclear genome of *M.homosphaera*, harboring 6649 protein-coding genes, is more compact than the genomes of the closely related Sphaeropleales species. This genome streamlining may be caused by a reduction in gene family number, intergenic size and introns. The genome sequence of *M.homosphaera* reveals the strategies adopted by this organism for environmental adaptation in the eutrophic lake. Analysis of cultures and the protein complement highlight the metabolic flexibility of *M.homosphaera*, the genome of which encodes genes involved in light harvesting, carbohydrate metabolism, and nitrogen and microelement metabolism, many of which form functional gene clusters. Reconstruction of the bioenergetic metabolic pathways of *M.homosphaera*, such as the lipid, starch and isoprenoid pathways, reveals characteristics that make this species suitable for biofuel production.

**Conclusion:**

The analysis of the whole genome of *M. homosphaera* provides insights into the genome streamlining, the high lipid yield, the environmental adaptation and phytoplankton evolution.

## Background

As the most urbanized and developed region of China, lake eutrophication is common in the middle-lower reaches of the Yangtze River. Picophytoplankton (with cell diameters < 3 μm) are abundant and can contribute 9–55% of primary productivity in eutrophic lakes [1, 2]. *Mychonastes* species are the dominant eukaryotic picophytoplankton in most eutrophic lakes (e.g., Lake Chaohu and Lake Poyang in China) [2, 3]. However, the mechanism underlying the dominance of *Mychonastes* in eutrophic lakes is not clear. Using a whole-genome approach, we specifically focused on the gene sets and metabolic pathways of *Mychonastes* that may facilitate its dominance under the environmental conditions of most eutrophic lakes [4, 5]. Although given the decreasing cost of sequencing [6–8], many phytoplankton have been sequenced [9–12], the genome sequencing of picophytoplankton has only targeted marine species thus far [13, 14]. The absence of genome information for picophytoplankton in freshwater lakes prevents us from recognizing the picophytoplankton niche and its ecological role in the lake.

*Mychonastes* belong to the order Sphaeropleales within the class *Chlorophyceae*. Sphaeropleales is a large group that contains some of the most common freshwater algae [15]. The genome sequences of Sphaeropleales are a hot research topic because some of these species show enormous potential for biofuel production [10, 11, 16], with robust growth and a high lipid content. Thus far, six genomes of Sphaeropleales, belonging to *Scenedesmus quadricauda* [9], *Raphidocelis subcapitata* [10], *Monoraphidium neglectum* [11], *Tetradesmus obliquus* [12], *Chromochloris zofingiensis* [17], and *Coelastrella* sp. [18], have been sequenced. These Sphaeropleales genomes provide much information for *Mychonastes* genome research and contribute to explaining the evolution and adaptation of *Mychonastes*. Comparative analyses of genomes would provide insights into the environmental adaptation and genome evolution of Sphaeropleales.

In order to further increase knowledge about the evolution and adaptation of freshwater picophytoplankton, we isolated a *Mychonastes* strain from Lake Chaohu, a highly eutrophic lake, and sequenced its complete genome by using third-generation sequencing (PacBio Sequel). Here, we conducted combined analysis of the complete genome sequences of *M.homosphaera* and other Sphaeropleales species as well as picophytoplankton species to investigate the evolutionary history and environmental adaptation of *M.homosphaera*.

## Results

### Phylogenetic analyses

**Fig. 1:**
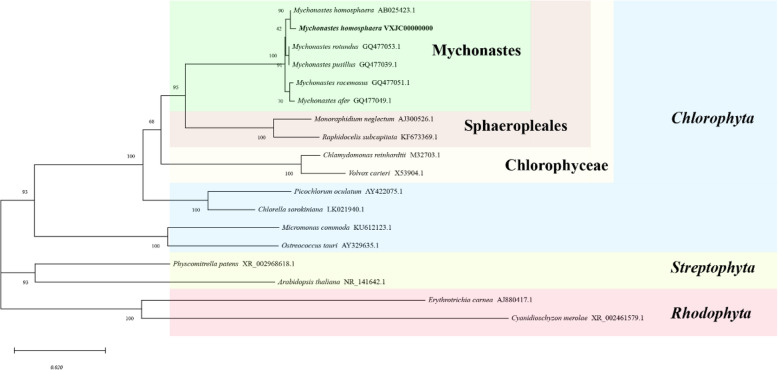
Phylogenetic tree of 18S rDNA sequences using the maximum likelihood method

We performed phylogenetic analyses using 18S rRNA to verify the phylogenetic position of *M.homosphaera* within Viridiplantae, with red algae as an outgroup (Fig. 1). In the tree, *M.homosphaera* was clustered by family, forming a monophyletic group with the other *Mychonastaceae* species. There was robust support (BP = 95) for the inclusion of *M.homosphaera* in *Mychonastaceae*, where it was positioned closest to *Mychonastes homosphaera* (AB025423) isolated from Lake Kinneret, Israel [19].

### General features of the nuclear genome

We sequenced 5.8 Gbp reads using the PacBio Sequel system. Based on assembly and correction, we obtained *M.homosphaera* genome statistics (genome size: 24.23 Mb, contig N50: 2 Mb, contig number: 31) (Table 1). The assembly was analyzed regarding its completeness based on sequence homology to the OrthoDB eukaryote dataset (www.orthodb.org), showing 89.4% complete BUSCOs (Benchmarking Universal Single-Copy Orthologs) (Supplementary Table 1), which was higher than the percentages for the sequenced Sphaeropleales species (*C.zofingiensis* 84.5%, *M.neglectum* 58.5%, and *T.obliquus* 79.9%) except for *R.subcapitata* (91.7%) [10]. Therefore, we obtained a nearly complete genome for *M.homosphaera.*

**Table 1:** *Mychonastes homosphaera* genome statistics

**Assembly statistics for the nuclear genome**	
Assembly genome size (Mbp)	24.23
Genomic G + C content (%)	72.4
Contig number	31
Number of Contig N50	5
Length of Contig N50 (kbp)	2001
miRNA number	0
rRNA number	26
snRNA number	11
tRNA number	64
**Gene statistics**	
Predicted number of nuclear genes	6649
Number of annotated genes	5711 (85.89%)
Average transcript length (bp)	2952.98
Average CDS length (bp)	1569.72
Exon number	32,277
Average exon number per gene	4.85
Average exon length (bp)	323.36
Intron number	25,628
Average intron number per gene	3.85
Average intron length (bp)	358.88
Coding (%)	43.1%

A total of 53,016 SSRs (simple sequence repeats) were masked by MISA (MIcroSAtellite identification tool), which accounted for 20.13% of the *M.homosphaera* genome. There were six types of SSR in the *M.homosphaera* genome (Supplementary Table 2), and the vast majority of SSR (52,206 repeat sequences) belong to those three types, p1, p2 and p3. Noncoding RNA in the genome was annotated differently; 26 rRNAs (including 6 18S rRNAs, 7 28S rRNAs, 6 5.8S rRNAs, and 7 5S rRNAs), 46 tRNAs and 11 snRNA were annotated. A total of 6649 protein-coding genes were predicted in the genome, with an average transcript length of 2952.98 bp and an average CDS (coding sequence) length of 1569.72 bp. Out of these, 5711 protein-coding genes (85.89% of the predicted genes) were annotated, and coding sequences constituted 43.1% of the genome, with a mean exon length and mean intron length of 323.36 and 358.88 bp, respectively. The protein-coding genes contained 25,628 introns, with a density of 3.85 introns per gene, and 32,277 exons, with a density of 4.85 introns per gene.

**Fig. 2:**
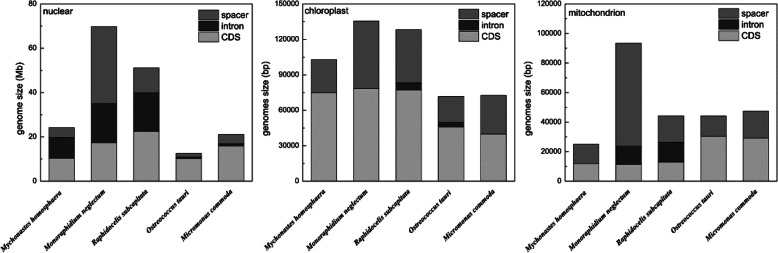
Size distributions of nuclear and organellar genomes of *M.homosphaera*, two Sphaeropleales species (*M.neglectum* and *R.subcapitata*) and two picophytoplankton species (*O.tauri* and *M.commoda*)

The nuclear genome of *M.homosphaera* was the smallest among those known for Sphaeropleales, at less than half of the size of the known whole genome sequences from Sphaeropleales. Unlike other Sphaeropleales species, *M.homosphaera* exhibited small intergenic regions and a high coding rate, which is common in other picophytoplankton (Fig. 2); therefore, the coding percentage of *M.homosphaera* (43.1%) was higher than that of other Sphaeropleales (expect *R.subcapitata*). Furthermore, *M.homosphaera* exhibited the highest GC content (72.4%) among the Sphaeropleales species examined to date.

### General features of chloroplast and mitochondrial genomes

**Fig. 3:**
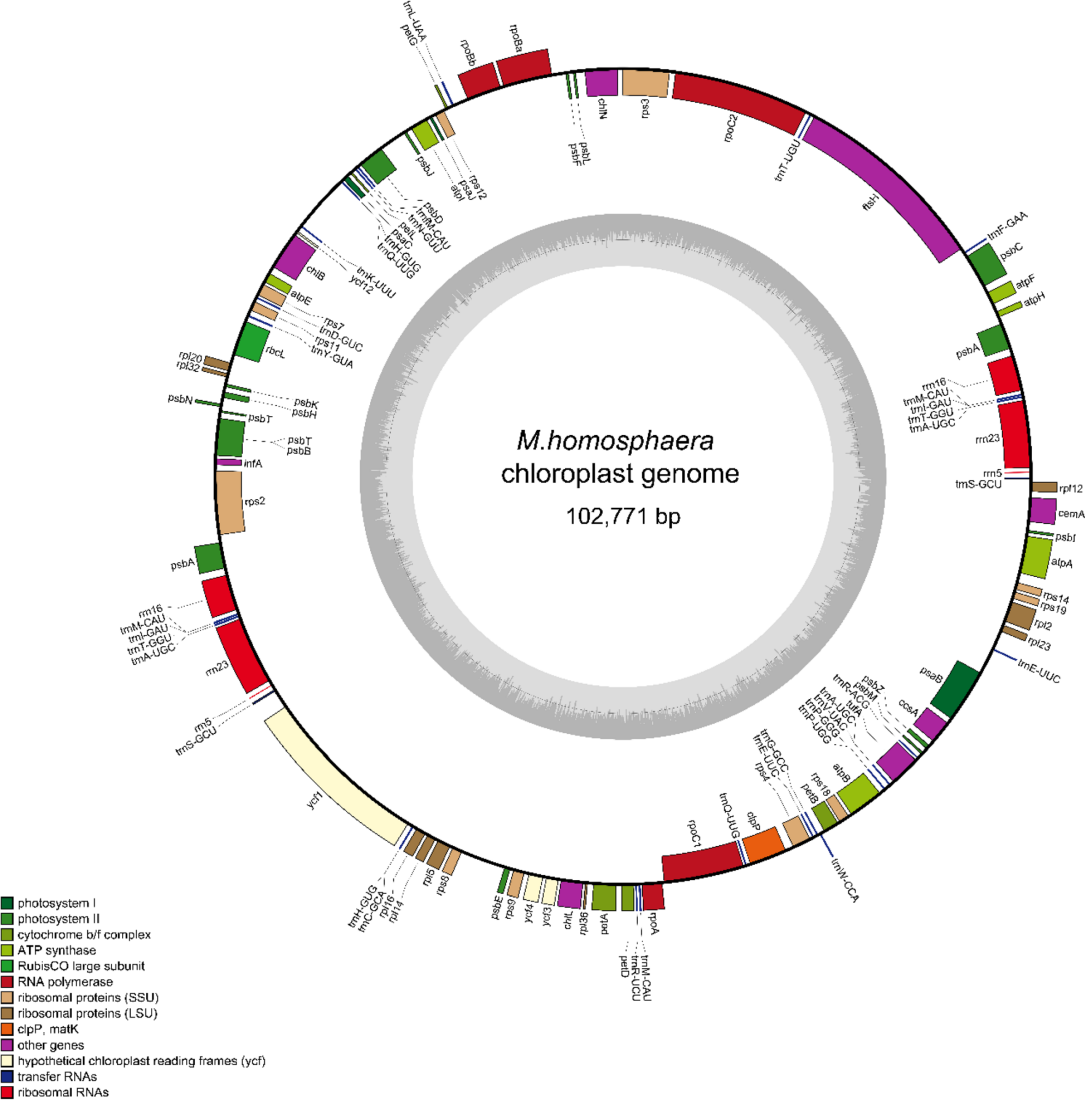
Chloroplast genome of *M.homosphaera*

*M.homosphaera* is the Sphaeropleales picophytoplankton, we compared its organelle genomes with those of other Sphaeropleales species (*M.neglectum* and *R.subcapitata*) and those of two marine picophytoplanktons (*Ostreococcus tauri* and *Micromonas commoda*), to understand the genome features of *M.homosphaera*. The complete chloroplast genome of *M.homosphaera* was one of the smallest among Sphaeropleales species identified thus far (102,771 bp in size, approximately two-thirds the size in other Sphaeropleales species), and it was AT-rich (60.03%) and circular with no inverted repeats or introns (Figs. 2 and 3). Surprisingly, *M.homosphaera* exhibited the maximum number of chloroplast genes among known Sphaeropleales, including 72 conserved protein-coding genes, 6 rRNAs and 35 tRNAs. Intronic ORFs (open reading frames) were not found in the chloroplast genome. Compared with other Sphaeropleales species, *M.homosphaera* presented extra rpl32 and apoprotein A1 genes (Supplementary Table 3). However, in fact, the CDS length of *M.homosphaera* was similar to those of other Sphaeropleales species.

**Fig. 4:**
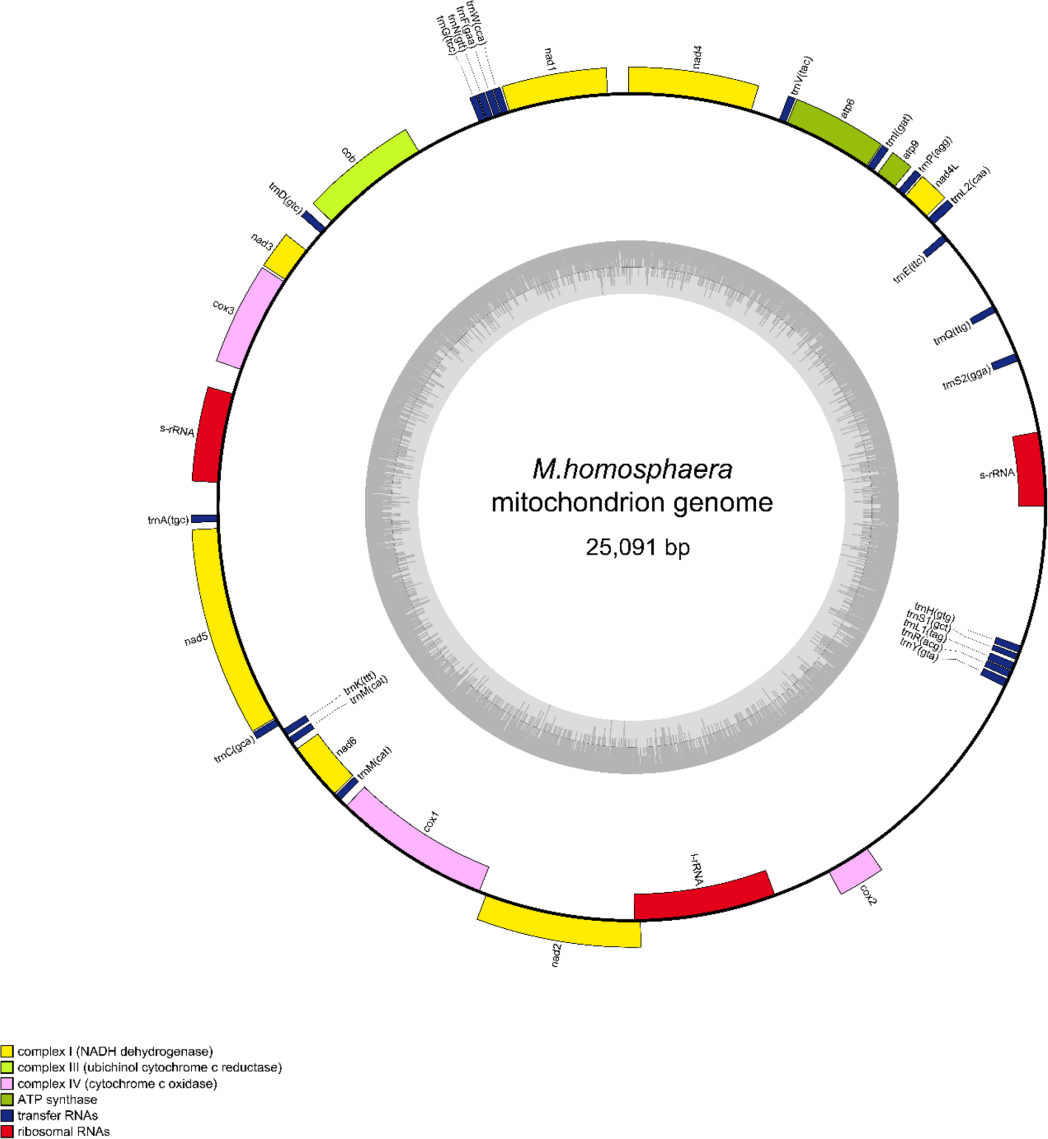
Mitochondrial genome of *M.homosphaera*

Extreme gene compaction was also founded in the mitochondrion (25,091 bp, 20.7% GC) (Figs. 2 and 4), which presented the smallest mitochondrial genome with the highest protein coding density identified to date within Sphaeropleales species while retaining the same genes found in other species (Supplementary Table 4). There were 13 conserved protein-coding genes, 6 fragmented rRNAs, and 22 tRNAs. The protein-coding genes included subunits of NADH dehydrogenase (nad1, nad2, nad3, nad4, nad4L, nad5 and nad6), ubichinol cytochrome c reductase (cob), cytochrome oxidases (cox1, cox2a and cox3) and 2 ATP synthases (atp6 and atp9). Similar with other Sphaeropleales species, the 16S rRNA and 23S rRNA sequences were separated into two and four fragments, respectively. Only a threonine-tRNA gene was missing in the mitochondrial genome, and there was an almost complete set of tRNAs for translation. In addition, we found that Cox2 was split; its N-terminus (Cox2a) was encoded by the mitochondrial genome, and the C-terminus of Cox2 (Cox2b) was encoded by the nuclear genome. Unlike other Sphaeropleales species, there were no introns in the *M.homosphaera* mitochondrial genome. However, the lack of introns had also been found in the mitochondrial genome of picophytoplankton such as *Ostreococcus tauri* and *Micromonas commoda* [13, 14] (Fig. 2)*.*

### Gene families of the *M.homosphaera* genus

**Fig. 5:**
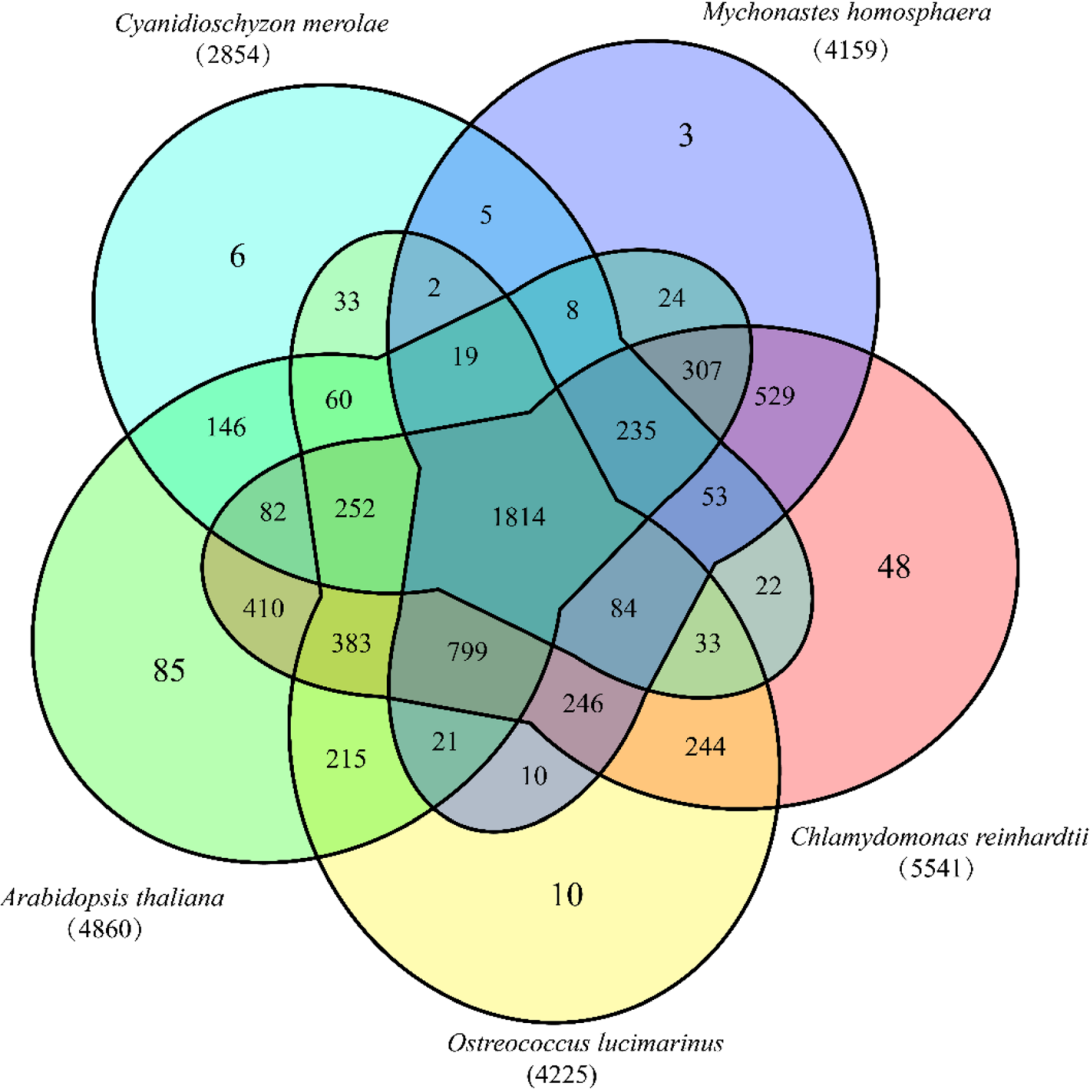
Venn diagram of the gene families of *M.homosphaera* and other Viridiplantae

To infer gene families variation in *M.homosphaera* in evolution, we compared its homologous genes with those of model organism, such as the red algae *Cyanidioschyzon merolae*, the green plant *Arabidopsis thaliana*, and two green algae (*O.tauri* and *Chlamydomonas reinhardtii*). The number of common gene families was 1814, accounting for approximately half of the *M.homosphaera* gene families (Fig. 5). Almost all of *M.homosphaera* gene families could be found in plants and algae, implying the evolutionarily ancient divergence of Plantae (red algae, green algae, and plants) [20]. In accord with the evolutionary direction, 529 gene families were shared by *M.homosphaera* and the green alga *C.reinhardtii*, whereas 24 and 5 gene families were only shared by *M.homosphaera* with *Arabidopsis thaliana* and *C.merolae*, respectively.

**Fig. 6:**
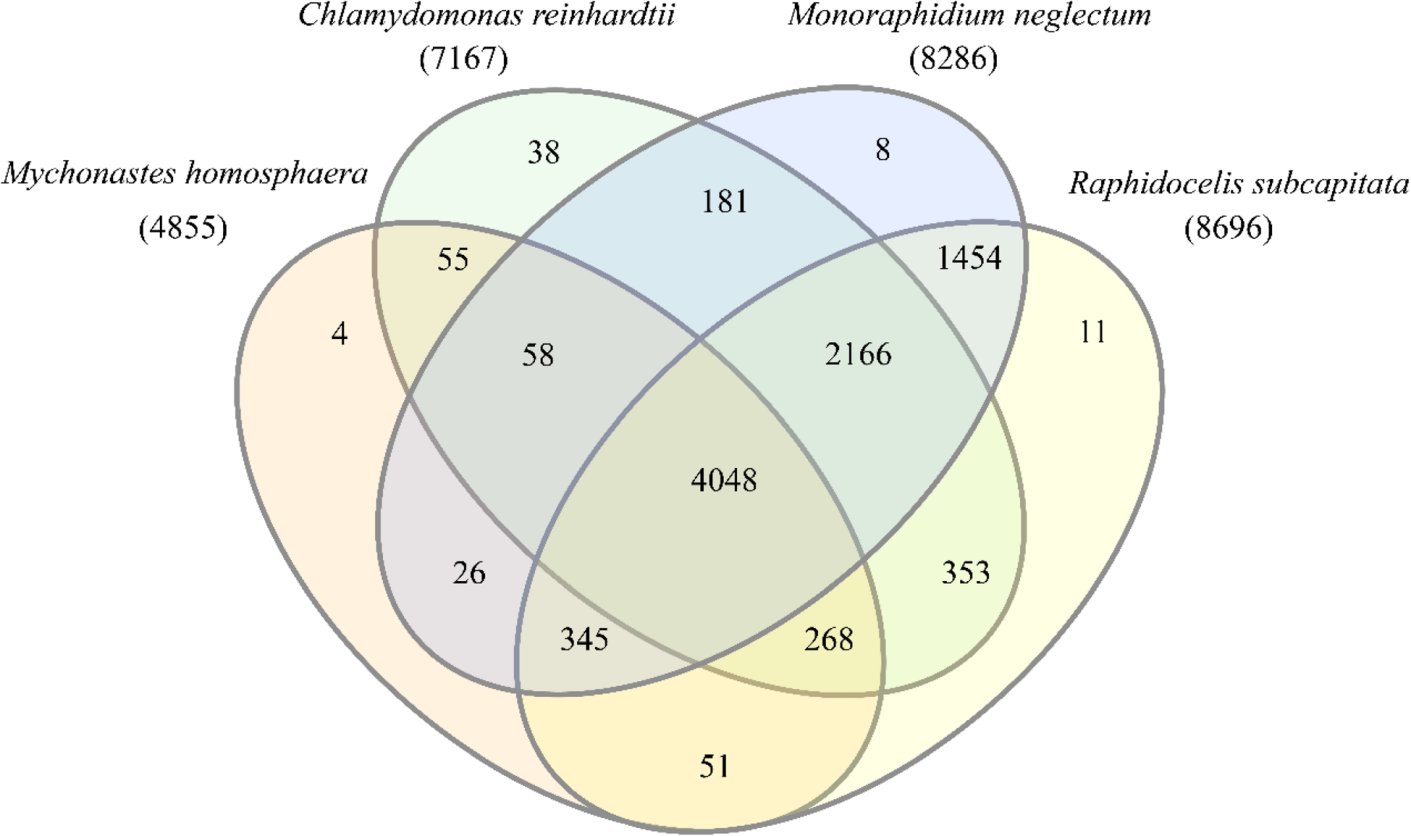
Venn diagram of the gene families of *M.homosphaera*, two Sphaeropleales species (*M.neglectum* and *R.subcapitata*) and a chlorophyte species (*C.reinhardtii*)

**Fig. 7:**
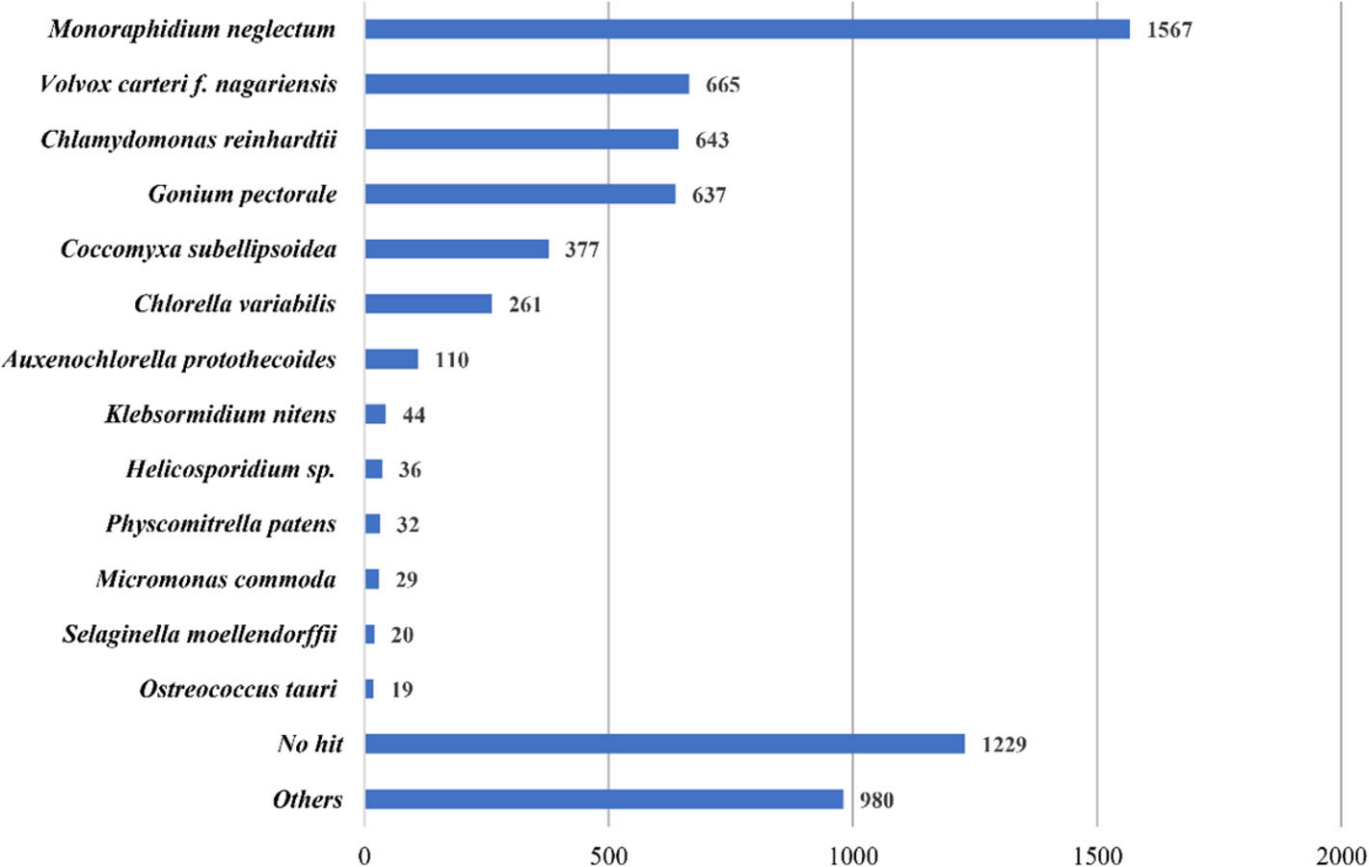
Top BLASTp hits of *M.homosphaera* compared with the nonredundant protein database

A similar comparison in green algae including Sphaeropleales species (such as *M.neglectum*, *R.subcapitata* and *M.homosphaera*) and *C.reinhardtii* was also performed (Fig. 6). The common numbers of gene families for green algae (*M.neglectum*, *R.subcapitata*, *M.homosphaera* and *C.reinhardtii*) was 4048, and for Sphaeropleales (*M.neglectum*, *R.subcapitata* and *M.homosphaera*) was 4393. *M.homosphaera* showed a lack of unique gene families, and more than 90 % of its gene families were common gene families. In addition, comparison of *M.homosphaera* genes to the nonredundant protein database yielded top hits from a variety of organisms, among which the highest frequency was found for the species *M.neglectum* and the taxon Chlorophyta (Fig. 7), which was expected on the basis of the phylogeny of *M.homosphaera.*

### Genome annotation and insights from the genome

**Fig. 8:**
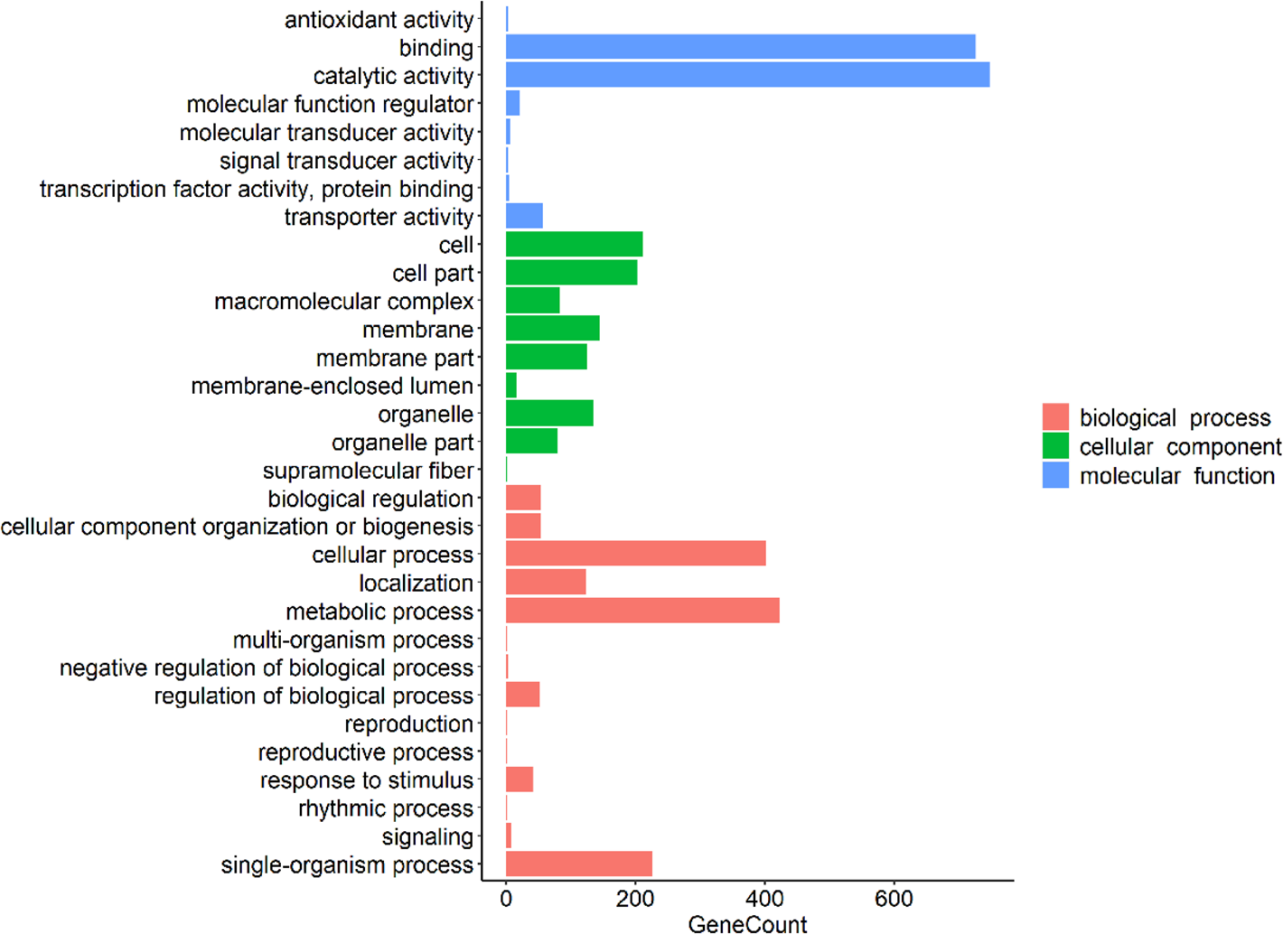
Gene Ontology (GO) assignments for *M.homosphaera* The 31 most extensive GO terms of the three GO supercategories “molecular function” (blue), “cellular component” (green) and “biological process” (red) are shown

The functions of 5711 proteins were predicted in the biochemical pathways of *M.homosphaera*, among which 3948 proteins were annotated based on homology with proteins in public databases. Furthermore, the annotated proteins were divided into functional categories based on the GO (Gene Ontology) database. The predicted proteins in *M.homosphaera* genome were divided based on three GO domains: molecular function, cellular component and biological process (Fig. 8).

**Fig. 9:**
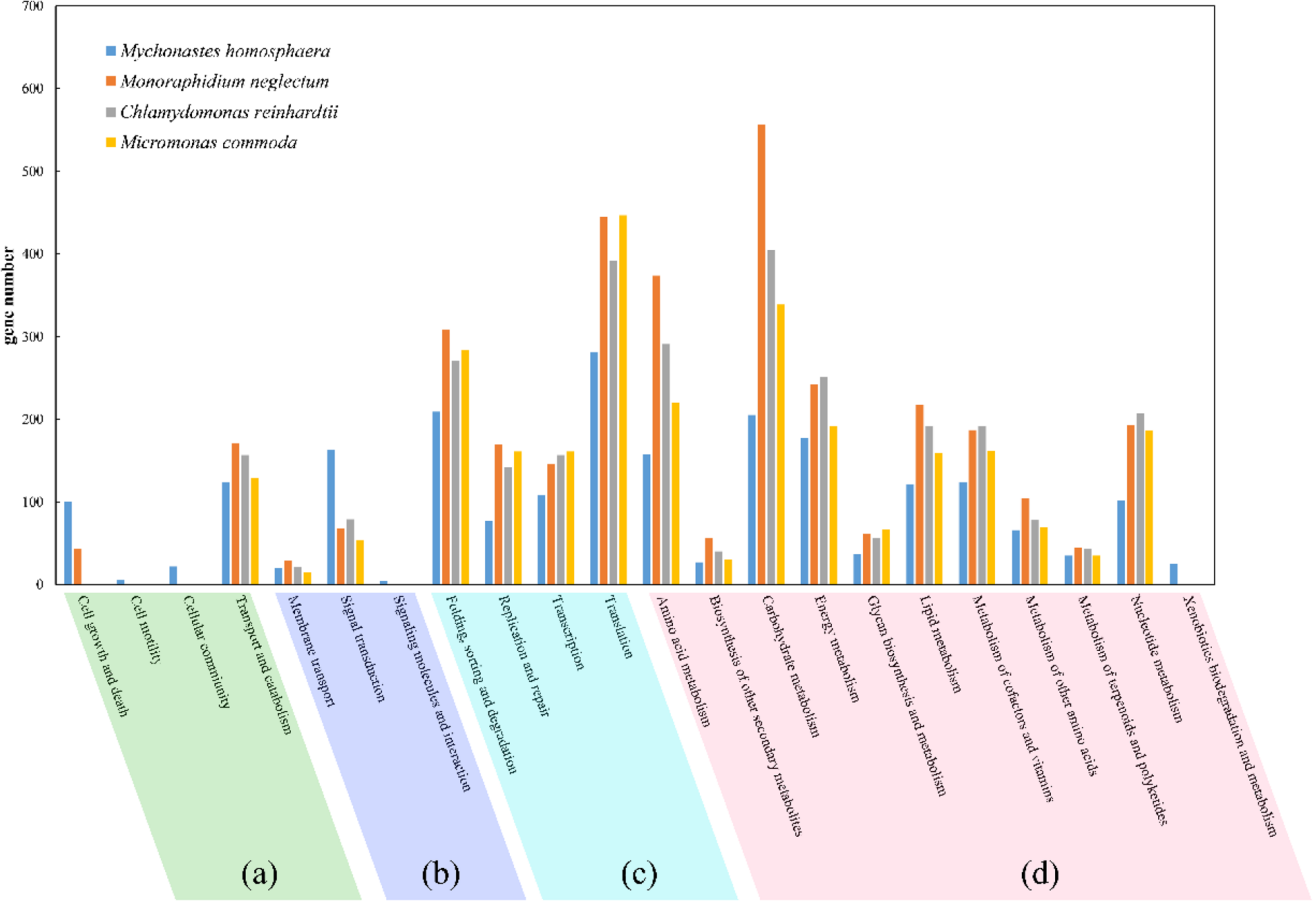
Functional comparison of *M.homosphaera* and other phytoplankton according to KEGG classification. (**a**), (**b**), (**c**) and (**d**) represent cellular processes, environmental information processing, genetic information processing and metabolism, respectively

Functional analyses using KEGG (Kyoto Encyclopedia of Genes and Genomes) categories showed that most of the functions were shared among phytoplankton, although *M.homosphaera* possessed the minimum number of genes among phytoplankton families (Fig. 9). *C.reinhardtii*, *M.neglectum* and *M.commoda* represent the chlorophyte, Sphaeropleales and picophytoplankton, respectively. However, the number of total genes in *M.homosphaera* were quite similar to those in other algae. Though the proportion of *M.homosphaera* genes related to various types of metabolism was relatively small, it possessed genes related to xenobiotic biodegradation, which are lacking in other algae. *M.homosphaera* contained a higher proportion of genes related to environmental information processing than other algae, especially signal transduction genes. Furthermore, *M.homosphaera* possessed all cellular process pathway genes, while *M.neglectum* possess transport, catabolism, cell growth and death pathway genes, and *C.reinhardtii* and *M.commoda* only possess transport and catabolism pathway genes.

### The genes of *M.homosphaera* facilitate its dominance within the environmental conditions of the lake

*M.homosphaera* are widely distributed in shallow turbid lakes and river-connected eutrophic lakes, experiencing complex and changing environmental conditions such as varying water exchange rates, light conditions, temperatures and nutrition concentrations. Genome analysis provides insights into the mechanism for adaptation to these environmental conditions.

#### Light Harvesting

**Fig. 10:**
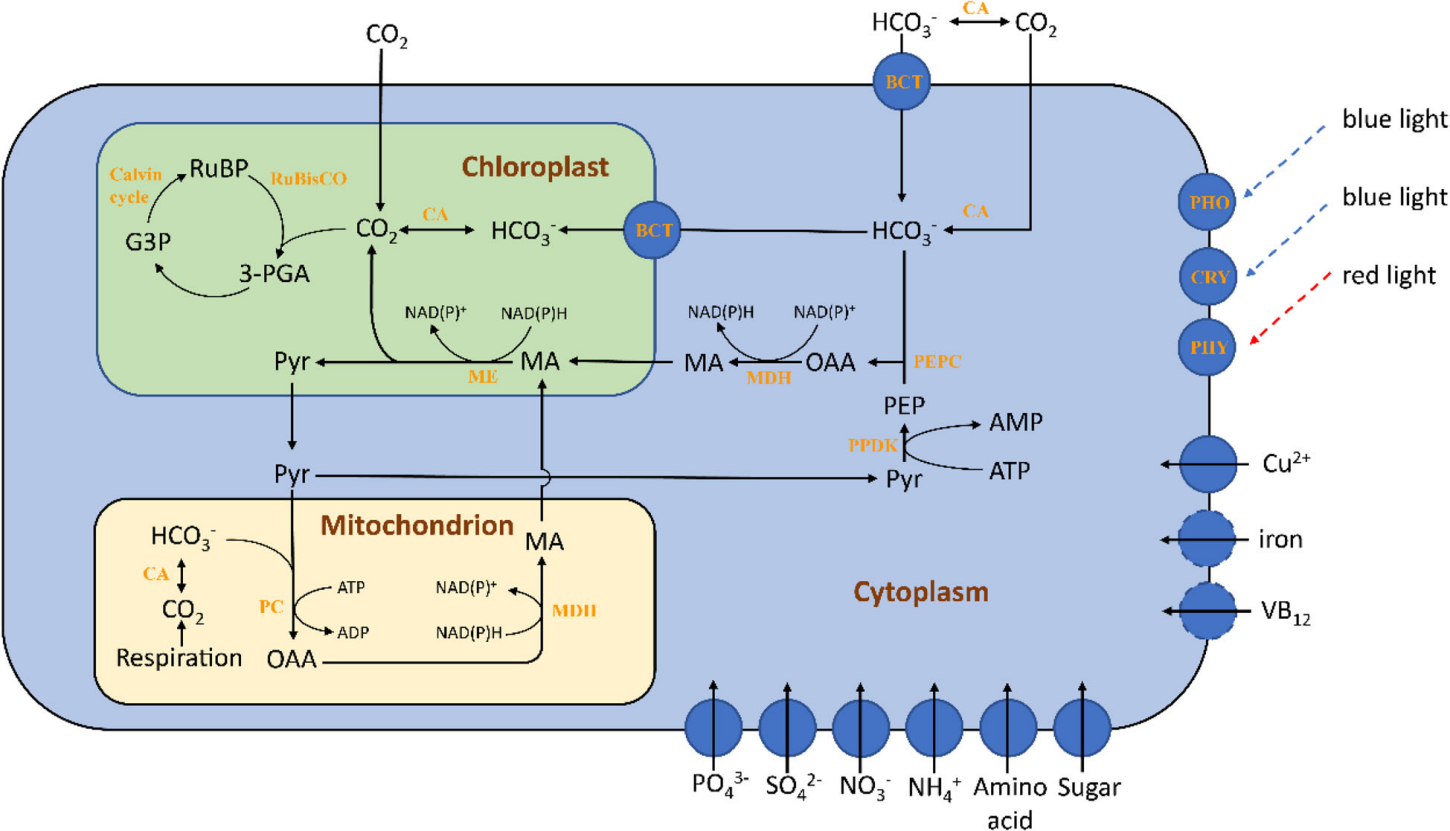
Carbohydrate metabolism, nutrient transport and photoreceptors in *Mychonastes homosphaera.* The metabolites are shown in black, and the enzymes are shown in yellow. G3P: glyceraldehyde 3-phosphate, MA: malic acid, OAA: oxaloacetate, PEP: phosphoenolpyruvate, 3-PGA: 3-phosphoglycerate, Pyr: pyruvate, RuBP: ribulose-1,5-bisphosphate, BCT: bicarbonate transporter, CA: carbonic anhydrase, MDH: malate dehydrogenase, ME: malic enzyme, PC: pyruvate carboxylase, PEPC: phosphoenolpyruvate carboxylase, PPDK: pyruvate, phosphate dikinase, RuBisCO: ribulose-1,5-bisphosphate carboxylase oxygenase, PHO: phototropins, CRY: cryptochromes, PHY: phytochrome. In nutrient transport, a solid line or circle indicates that the gene has been identified, and a dashed circle indicates that the gene may be present. Metabolic pathway reconstruction was performed based on the KEGG database

*M.homosphaera* possessed genes homologous to phototropins and cryptochromes, which generally act as blue-light photoreceptors in certain eukaryotes. A phytochrome gene homolog was also identified; phytochromes function as red/far-red light and temperature sensors. However, there were no rhodopsin green-light photoreceptors in *M.homosphaera* (Fig. 10).

The xanthophyll cycle is a major mechanism for the dissipation of excess light energy for plant photoprotection [21, 22]. Two key enzymes of the xanthophyll cycle, VDE (violaxanthin de-epoxidase) and ZE (zeaxanthin epoxidase), can be found in *M.homosphaera.* In addition, Fe-Mn SOD (superoxide dismutase)-encoding genes were found. Furthermore, the *M.homosphaera* genome contained the full suite of genes involved in photosynthesis, including 13 genes encoding components of the LHC (light-harvesting complex), which absorbs light and transfers it to photosynthetic reaction centers.

#### Carbohydrate metabolism

Carbon concentrations and carbon assimilation have been described in many algae [20, 23, 24], and we were able to identify the genes required for both C3 (Calvin cycle)- and C4-type carbon assimilation (Supplementary Table 5). The key enzymes in these two types of metabolism are carbonic anhydrases, which catalyze the reversible conversion of CO_2_ to HCO_3_^−^. In addition, HCO_3_^−^ must be transported into the cell by a bicarbonate transporter or be converted into CO_2_ by carbonic anhydrases. These two enzymes were found in *M.homosphaera* genome (Fig. 10).

Based on the obtained protein information and the Randor model [24], we describe potential carbon-concentrating mechanisms in *M.homosphaera* (Fig. 10). There are two potential C4 carbon-concentrating mechanisms, which involve the production of malate in the cytosol and the mitochondria, and they provide possibilities for carbon concentration under different environmental conditions.

#### Nitrogen assimilation

Nitrogen is the most common nutrient limiting primary production in freshwater, estuarine and coastal ecosystems. *M.homosphaera* possessed genes encoding nitrate, nitrite, and ammonium transporters, which indicates that *M.homosphaera* uses multiple forms of nitrogen (Supplementary Table 6). Additionally, the *M.homosphaera* genome encoded one NAD(P)H-nitrate reductase, for the reduction of nitrate to nitrite, and one ferredoxin nitrite reductase, to catalyze the reduction of nitrite. Additionally, the *M.homosphaera* genome encoded all urea cycle components except for arginase, including carbamoyl-phosphate synthetase I (large and small subunits), ornithine carbamoyltransferase, arginosuccinate synthase, and arginosuccinate lyase.

#### Microelements

*M.homosphaera* lacked all genes for common iron acquisition, although iron is involved in many metabolic activities, such as photosynthesis, respiration and nitrate reduction. Therefore, there may be a novel iron acquisition system in *M.homosphaera* that is different from those of other phytoplankton. The lack of iron transport components is also found in other picophytoplankton, such as *Ostreococcus* [25], which also possesses a small genome size and gene number. Plants generally utilize copper transporter (CTR) family proteins to transport Cu ions into the cytosol [26], and these proteins were not found in *M.homosphaera.* However, there were two ZIP (Zrt, Irt-like Protein) genes in this organism, which are speculated to function in Cu acquisition, perhaps showing the capacity to transport Cu^2+^ [27].

In methionine synthesis, in addition to the VB_12_ (vitamin B12)-dependent pathway, there are VB_12_-independent pathways in phytoplankton [28]. However, *M.homosphaera* exhibited METH (methionine synthase) but not METE (B_12_-independent methionine synthase), indicating that it only performs methionine synthesis via the VB_12_-dependent pathway. Therefore, *M.homosphaera* shows strict dependence on VB_12_. VB_12_ can only be produced by bacteria (both eubacteria and archaea) in nature [29]. Therefore, *M.homosphaera* must acquire VB_12_ or a precursor from the lake environment or associated bacteria [30]. In addition, the *M.homosphaera* genome possessed a complete pathway for thiamine biosynthesis (Supplementary Table 7), which increases biotic and stress resistance [31, 32].

### Bioenergetic metabolic pathway reconstruction in *M.homosphaera*

#### Lipid metabolism

**Fig. 11:**
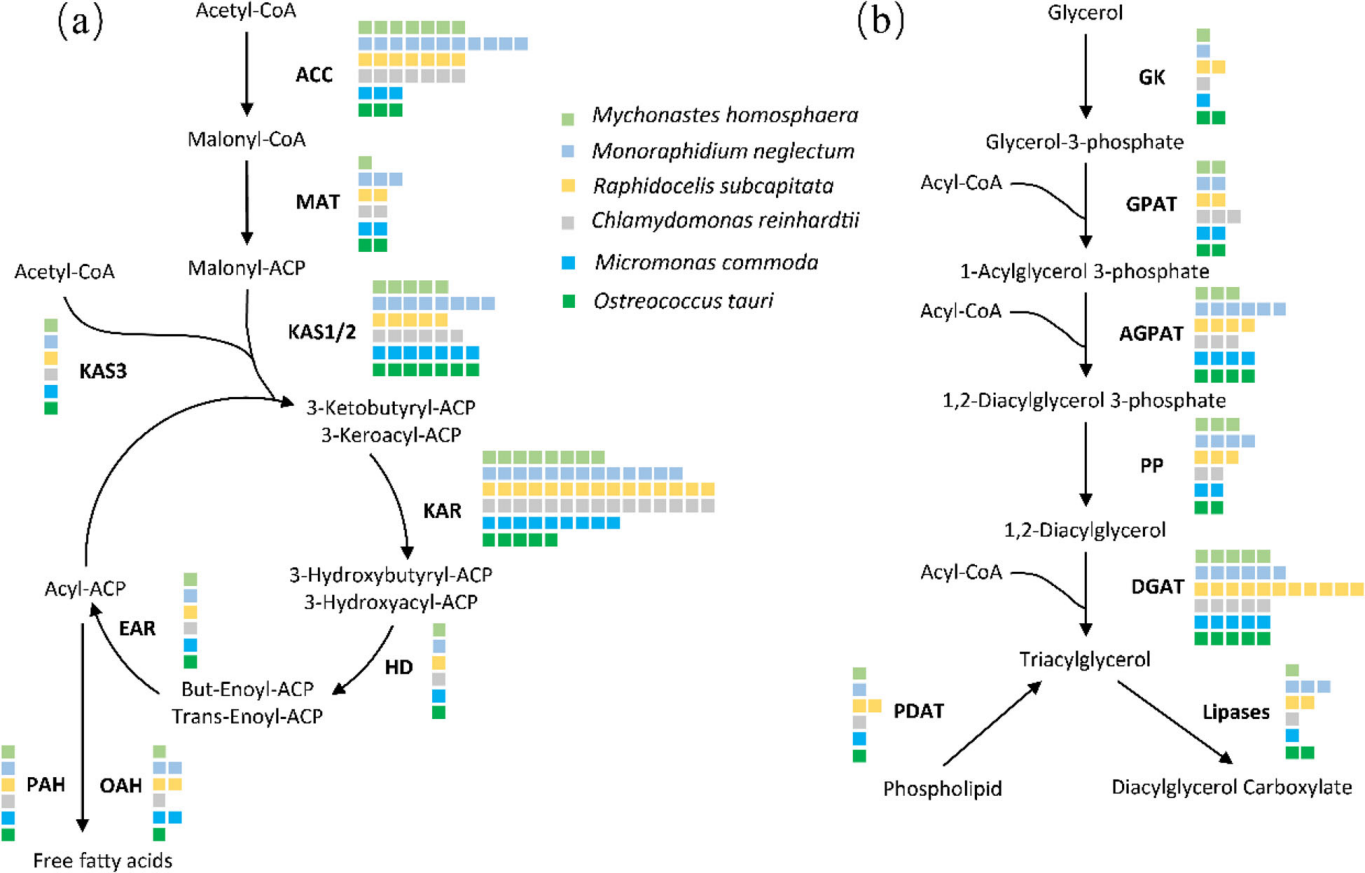
Fatty acid biosynthesis pathways (**a**) and the TCA (glycerolipid) metabolism pathways (**b**) of *Mychonastes homosphaera*. ACC: acetyl-CoA carboxylase, MAT: malonyl-CoA:ACP transacylase, KAS3: the beta-ketoacyl-acyl-carrier protein synthase 3, KAS1/2: the beta-ketoacyl-acyl-carrier-protein synthase, KAR: the 3-oxoacyl-ACP reductase, HAD: the beta-hydroxyacyl-ACP dehydrase, EAR: enoyl-ACP reductase, OAH: oleoyl-acyl-carrier-protein hydrolase, PAH: palmitoyl-protein thioesterase, GK: glycerol kinase, GPAT: glycerol-3phosphate O-acyltransferase, AGPAT: 1-acylglycerol-3phosphate O-acyltransferase, PP: phosphatidate phosphatase, DGAT: acyl-CoA:diacylglycerol acyltransferase, PDAT: phospholipid:diacylglycerol acyltransferase. Each coloured square represents a homologous gene, and different color represent different species. Pathway reconstruction was performed for fatty acid biosynthesis and TCA synthesis based on the KEGG database

Many microalgae, especially Sphaeropleales species, have been reported to produce considerable amounts of biofuels [10, 11, 16]. Therefore, lipid metabolism pathway reconstruction in *M.homosphaera* provides essential new insights into the lipid metabolism of phytoplankton and provides a basis for further investigations and genetic improvements. We compiled the genes related to lipid metabolism using the KEGG database to reconstruct the fatty acid biosynthesis pathways and TCA (glycerolipid) metabolism pathways of *M.homosphaera.* Additionally, we reconstructed the lipid metabolism pathways of other phytoplankton in the same way and compared them with those of *M.homosphaera* (Fig. 11 and Supplementary Table 8).

*M.homosphaera* possesses a similar number of genes to other phytoplankton despite its small genome size, and the key fatty acid biosynthesis pathways have been identified. Notably, *M.homosphaera* exhibited a relatively high number of homologous genes for ACC (acetyl-CoA carboxylase, seven genes) and KAR (3-oxoacyl-ACP reductase, 11 genes). This situation also exists in other Sphaeropleales species.

Furthermore, the glycerolipid metabolism pathways of *M.homosphaera* were the same as those of other phytoplankton despite the different genome sizes. TAG (triacylglycerol) can be formed through glycerolipid metabolism via acylCoA–independent or dependent pathways catalyzed by PDAT (phospholipid: diacylglycerol acyltransferase) and DGAT (acyl-CoA:diacylglycerol acyltransferase), respectively. DGAT catalyzes the final step in the acylCoA–dependent pathway, leading to TAG. The DGAT gene family, including DGAT1 and DGAT2, was identified in the past decade [33, 34]. DGAT2 was present in all the phytoplankton that we compared; however, DGAT1 only existed in *M.homosphaera* and other Sphaeropleales species. PDAT is a key enzyme in the acylCoA–independent pathway and is able to hydrolyze not only phospholipids, cholesteryl esters and galactolipids but also TAG. Therefore, PDAT plays an important role in membrane turnover as well as TAG synthesis and degradation. Although the total genome sizes of *M.homosphaera* and other picophytoplankton are significantly lower than those of other phytoplankton, the numbers of genes in glycerolipid metabolism pathways are not significantly different between these groups.

#### Starch and isoprenoid metabolism

**Fig. 12:**
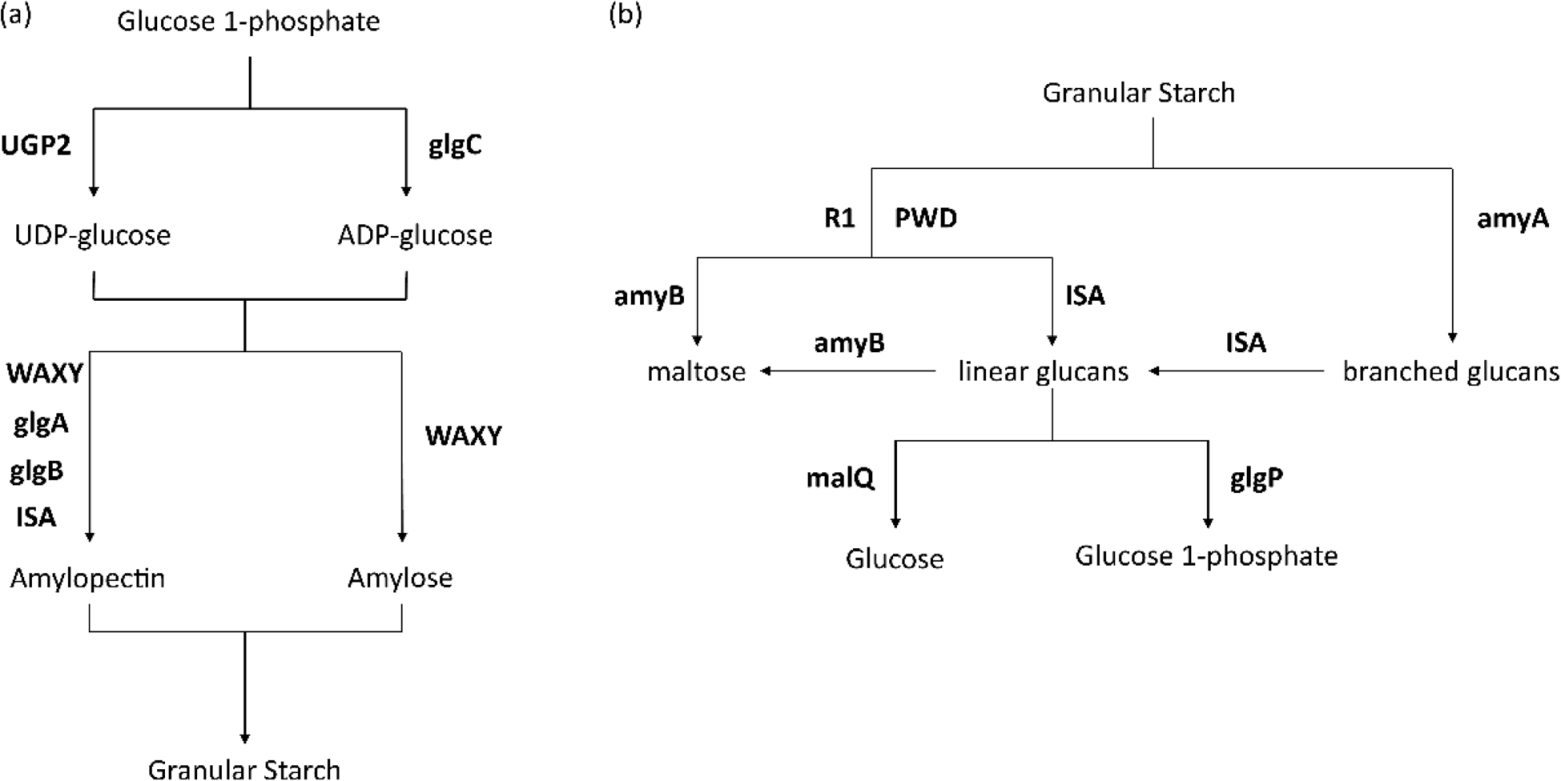
Starch synthesis (**a**) and degradation (**b**) in *Mychonastes homosphaera*. glgC: glucose-1-phosphate adenylyltransferase, WAXY: granule-bound starch synthase, glgA: starch synthase, glgB: glucan branching enzyme, ISA: isoamylase, R1: alpha-glucan, water dikinase, PWD: phosphoglucan, water dikinase, amyA: alpha-amylase, amyB: beta-amylase, malQ: 4-alpha-glucanotransferase, glgP: starch phosphorylase. Pathway reconstruction was performed for starch synthesis and starch degradation based on the KEGG database

We analyzed genes related to starch metabolism using the KEGG database and Busi’s research [35] to reconstruct the starch metabolism pathways of *M.homosphaera* (Fig. 12 and Supplementary Table 9). There are four biochemical steps in starch synthesis: substrate activation, chain elongation, chain branching, and chain debranching [36, 37].

There are two isoprenoid biosynthesis pathways found in organisms: the mevalonate (MVA) and the nonmevalonate (DXP) pathways [38]. Similar to other green algae, such as *C.reinhardtii, Scenedesmus obliquus* and *Ostreococcus lucimarinus*, *M.homosphaera* has abandoned the MVA pathway and retained only the DXP pathway (Supplementary Table 10).

## Discussion

### Environmental prevalence and adaptation of *M.homosphaera*

Understanding the response of this algae to a stressful and fluctuating environment offers the promise of advancing both applied and fundamental research. *Mychonastes* is a common chlorophycean picoplankton genus in freshwater ecosystems [39] and is widely found in lakes and rivers in Asia [3, 40–42], Europe [43] and North America [44]. *Mychonastes* species are small (< 3 μm) unicellular organisms (spherical, ovate and ellipsoid) surrounded by a cell wall, possessing one mitochondria and chloroplast. *Mychonastes* species are prevalent in river-connected lakes where diatoms occur [45], and recent research has shown that *Mychonastes* is the dominant eukaryotic picophytoplankton group in spring and winter in many eutrophic lakes, such as Lake Chaohu and Lake Poyang [2, 3, 40]. The whole genome of *M.homosphaera* could provide insight into its adaptive mechanism.

For phytoplankton, only a few resources are potentially limiting, e.g., light, nitrogen, phosphorus, inorganic carbon, silicon, iron,, and sometimes a few trace metals or vitamins [46]. *M.homosphaera* possesses many nutrient transport genes, such as genes encoding nitrogen, phosphorus, metal, and vitamin transporters, that may improve its nutrient uptake ability at a relatively low trophic level. In addition, *M.homosphaera* could use various sources of nitrogen compounds, strengthening its competitive ability under nutrient-depleted conditions. The presence of various potential carbon-concentrating mechanisms in *M.homosphaera* would provide more possibilities for carbon assimilation under different environmental conditions. Furthermore, for the supplementation of micronutrient such as metals and vitamins, *M.homosphaera* possesses its own acquisition pathway.

Light intensity is relatively low and becomes an important limiting factor for phytoplankton growth in spring and winter. *M.homosphaera* possesses gene homologs of phototropins, cryptochromes and phytochromes, which absorb red light and blue light [47]. Compared with its major counterpart taxon, the diatoms, *M.homosphaera* lacks rhodopsins, which absorb green light. Red light and blue light are both absorbed at relatively shallow depths, but green light usually penetrates the water column to greater depths than other wavelengths [48]. Thus, the presence of blue/red-light photoreceptors and the absence of green-light photoreceptors increases the adaptation of *M.homosphaera* to the shallow turbid lake and implies genome streamlining. In addition, the high abundance of light harvesting complex components in *M.homosphaera* contributes to its adaptation to the low light conditions found in Lake Chaohu in winter.

Light is essential for algae, but excess light will damage the growth of algae. Phytoplankton adjust their light absorption and eliminate excess light energy when light is abundant or excessive [49]. *M.homosphaera* possesses the light dissipation mechanism of the xanthophyll cycle, which provides the ability to remove accumulated harmful byproducts, such as three line states of chlorophyll molecules and singlet oxygen, resulting from excess light [50]. In addition, the existence of Fe-Mn SODs will decrease the damage caused by reactive oxygen generated by excess light [51].

The water temperatures of Lake Chaohu in winter are normally lower than 10 °C [40], which is a challenge for phytoplankton growth. To adjust to the winter conditions in the lake, *M.homosphaera* possesses many mechanisms to respond to low temperatures. *M.homosphaera* possesses a gene encoding a pyrroline-5-carboxylate reductase that can synthesize proline to alleviate osmotic stress caused by cold. *M.homosphaera* also possesses genes encoding antioxidases (such as Fe-Mn SOD, catalase, and ascorbate peroxidase) and antioxidant (such as glutamate and β-carotene) biosynthetic pathways. Both of these substances can remove toxic active oxygen derived from excess O_2_ and H_2_O_2_ in cells under cold stress [52–54]. Unsaturated fatty acids can reduce the phase transition temperature of the plant cytomembrane to prevent plants from being damaged by temperature stress [55–58]. *M.homosphaera* possesses genes encoding the synthesis of large fatty acids, especially unsaturated fatty acids. The genome sequence of *M.homosphaera* indicates enrichment of unsaturated fatty acids, which is consistent with other research (more than 70%) [59]. The high content of unsaturated fatty acids reduces the *M.homosphaera* phase transition temperature and improves the adaptation of this species in Lake Chaohu. In addition, phytochromes act as temperature sensors that integrate temperature information over the course of the night [60], as warm temperatures reduce the activation of phytochromes, which may improve the adaptation of *M.homosphaera* to temperature differences between day and night.

Copper deficiency can lead to the inhibition of organism growth, photosynthesis, and respiration. However, excessive copper also has obvious toxic effects on algal bodies, even leading to plant death [61, 62]. To adapt to an excessive copper environment, *M.homosphaera* may possess multiple mechanisms for ameliorating copper toxicity. *M.homosphaera* encodes a phytochelatin synthase that can chelate metal ions to reduce copper toxicity [63] and a Fe-Mn SOD to dispel the free radicals formed due to copper stress [64].

### The *M.homosphaera* genome reveals characteristics suitable for biofuel production

Algae are also very important because they synthesize a number of different lipids and carbon storage compounds (such as starch and isoprenoid) that possess high biological and commercial value [35]. These characteristics, including high biomass production rates, high starch content and TAG accumulation, make algae a possible biofuel resource. Compared with starch accumulation, Sphaeropleales may show an advantage in TAG accumulation [11].

The complete lipid pathways and abundant homologous gene family in the *M.homosphaera* genome indicate a high potential lipid yield. Furthermore, the quality of biodiesel is mainly determined by the composition of fatty acids [65]. The abundance of KCS (3-ketoacyl-CoA synthase) and FAD (fatty acid desaturase) homologous genes indicates that *M.homosphaera* tends to synthesize long-chain unsaturated fatty acids, which are generally well suited for biodiesel generation [66]. In addition, TAG accumulation in phytoplankton usually occurs under stress conditions such as high light or nitrogen starvation [67]. The TAG yield can also be increased artificially by inhibition of starch synthesis [68]. Laboratory experiments revealed that *M.homosphaera* could have a high growth rate even under environmental stress [69]. In addition to the high specific uptake rate of CO_2_, *M.homosphaera* is capable of utilizing polysaccharides as a carbon source. Therefore, extensive carbon sources, efficient photosynthesis, rapid growth and lipid abundance indicate that *M.homosphaera* could be a preferred source for biodiesel production. Environmental condition control and biotechnological applications to improve the yield and quality of TAG would make *M.homosphaera* more appropriate to biodiesel production.

### Genome streamlining of *M.homosphaera*

It is commonly believed that evolution generally proceeds towards increased complexity at both the genomic and organismal levels. However, recent evolutionary reconstructions indicate that genome reduction is a dominant mode of genome evolution, consistent with the optimization of initially highly complex large genomes, leading to the acquisition of adaptive innovations [70]. *M.homosphaera* and other picophytoplankton, such as *Ostreococcus tauri* and *Micromonas commoda,* possess smaller and more streamlined genomes than phytoplankton of the same genus. Streamlined genomes are characteristic of fast-evolving species that live in specialized ecological niches or in extreme environments [71]. The whole-genome analysis suggested that there are two routes for genome reduction in *M.homosphaera*, namely, the reduction of gene family number and of noncoding region (intergenic and intron) size. Although *M.homosphaera* possesses the minimum number of gene families in the order Sphaeropleales, this species has retained all the fundamental functional genes and can grow well in uni-algal laboratory experiments [69]. In addition, *M.homosphaera* possesses nearly the most dense nuclear genome (24.23 Mb) in Chlorophyceae, second only to *R*.*subcapitata*, with mean intergenic and intron lengths of 685 and 359 bp, respectively. This streamlining is particularly obvious in organelle genomes, including those of chloroplasts and mitochondria. *M.homosphaera* have the highest protein coding density among Sphaeropleales*,* and these genomes do not contain introns, leading to the reduction in organelle genomes. This compact nuclear genome has also been found in other picophytoplankton, such as *O.tauri* and *M.commoda*, although their genetic relationships are relatively distant. This result may indicate that the genome architecture is consistent with cell size in phytoplankton, although the relationship between the genome architecture and cell size remains controversial [72–74]. Such streamlined genomes are also detectable in the cyanobacterium *Prochlorococcus sp.* [75, 76] and the alpha-proteobacterium *Candidatus Pelagibacter ubique* [77–79], both of which are highly successful free-living organisms and apparently the most abundant cellular life forms on earth.

### *M.homosphaera* genomes provide insights into evolutionary processes

Previous research indicates that the algae in Sphaeropleales harbor split cox2 genes, including a mitochondrion-localized cox2a gene and a nucleus-localized cox2b gene [80]. Consistent with this finding, *M.homosphaera* harbored split cox2 genes, comprising a mitochondrion-localized cox2a gene and a nucleus-localized cox2b gene. However, Prasinophyceae, Ulvophyceae and Trebouxiophyceae contain orthodox, intact, mitochondrial cox2 genes, having evolved earlier than Chlamydomonadales, which possess both split cox2 genes in their nuclear genome [81]. Sphaeropleales show an intermediate trait of gene migration to the nucleus, which indicates that the appearance of Sphaeropleales occurred between that of Ulvophyceae and Chlamydomonadales.

In addition, the presence of NUMTs (nuclear mitochondrial DNA) and NUPTs (nuclear plastid DNA) (Supplementary Text, Supplementary Table 11 and 12) in *M.homosphaera* genomes verified gene migration from organelles to the nucleus. This is an important process for genome streamlining in most eukaryotes [82], as migrated genes are usually inserted into the intergenic space of the nuclear genome to improve the coding rate of the nuclear genome. During the evolution of mitochondria, many mitochondrial genes have been functionally transferred to the nucleus, whereas others have been replaced by preexisting nuclear genes which possessed similar function [83].

The Endosymbiotic Hypothesis is a hypothesis about the origins of mitochondria and chloroplasts, which are organelles of eukaryotic cells. According to this, land plants and green algae acquired their plastids from the same endosymbiotic event, the Glaucophyta and red algae (Rhodophyta), likely also originated from secondary endosymbiosis [84]. The DXP pathway is provided by the eukaryotic host cell during the primary endosymbiotic event in phototrophs with primary plastids. In contrast, the MVA pathway is contributed by the secondary eukaryotic host, with possible contributions from the primary host cell in algal groups emerging from secondary endosymbiosis [85]. Similar to other green algae, such as *C.reinhardtii, S.obliquus* and *O.lucimarinus*, *M.homosphaera* had abandoned the MVA pathway and retained only the DXP pathway [24]. However, the primary endosymbiotic algal groups Glaucophyta and Rhodophyta (except *C.merolae*) and secondary endosymbiotic algal groups Euglenophyta, Chlorarachniophyta, Haptophyta and Heterokontophyta have maintained both the MVA and DXP pathways. Therefore, the abandonment of the MVA pathway and the presence of the DXP pathway in *M.homosphaera* could support the endosymbiosis hypothesis.

## Conclusions

This study focused on the analysis of the whole genome of *M.homosphaera*, a eukaryotic picophytoplankton, providing insights into the environmental adaptation mechanism of these organisms, including efficient and comprehensive nutrient utilization capacity, a special light harvesting system to adapt to low light conditions, a multifarious defense mechanism against coldness, and various response mechanisms to improve resistance to environmental stress. In addition, *M.homosphaera* exhibits high lipid yields, particularly of long-chain unsaturated fatty acids. Therefore, this species is generally well suited for biodiesel generation. Similar to other picophytoplankton, streamlining and genome compaction was observed for *M.homosphaera* Streamlining of the genome of *M.homosphaera* may be caused by the reduction in gene family number and noncoding region size. With respect to genetic evolution, the split cox2 genes in *M.homosphaera* indicate gene migration from the organelles to the nucleus and the abandonment of the MVA pathway, and the presence of the DXP pathway in *M.homosphaera* could support the endosymbiosis hypothesis.

## Methods

### Isolation and culture of *M.homosphaera*

*M.homosphaera* was isolated from the central region Lake Chaohu (31.5294°N, 117.5261°E) in December 2015, and preserved in our laboratory at the Nanjing Institute of Geography and Limnology, Chinese Academy of Sciences. The strain was cultivated at 15 °C in BG11 medium under light (12 h light:12 h dark; 25 μmol photo m^− 2^ s^− 1^) in 250-mL glass flasks.

### DNA extraction

*M.homosphaera* was collected, and genomic DNA was extracted by using the QIAGEN Genomic DNA Extraction Kit (Cat# 13323, QIAGEN) according to the standard operating procedure provided by the manufacturer. The extracted DNA was detected by a NanoDrop™ One UV-Vis spectrophotometer (Thermo Fisher Scientific, USA) for DNA purity (OD260/280 ranging from 1.8 to 2.0 and OD 260/230 is between 2.0 and 2.2), and then, a Qubit 3.0 fluorometer (Invitrogen, USA) was used to quantify the DNA accurately.

### Library construction and sequencing

After the sample was qualitatively analyzed, the genomic DNA was sheared by using g-TUBEs (Covaris, USA) according to the expected sizes of the fragments for the library. The fragmented DNA of the target size was enriched and purified by using MegBeads. Next, the fragmented DNA was repaired for damage and then end-repaired. The stem-loop adaptor was linked on both ends of each DNA fragment, and the link-failed fragments were removed by exonuclease. Then, the target fragments were screened by BluePippin (Sage Science, USA) and purified to construct the library. Finally, an Agilent 2100 Bioanalyzer (Agilent Technologies, USA) was used to determine the sizes of the library fragments.

After the library was constructed, DNA templates and enzyme complexes of a certain concentration and volume were transferred to the ZMWs of the Sequel system (Pacific Biosciences, USA) for real-time single-molecule sequencing.

### Assembly and annotation

**Fig. 13:**
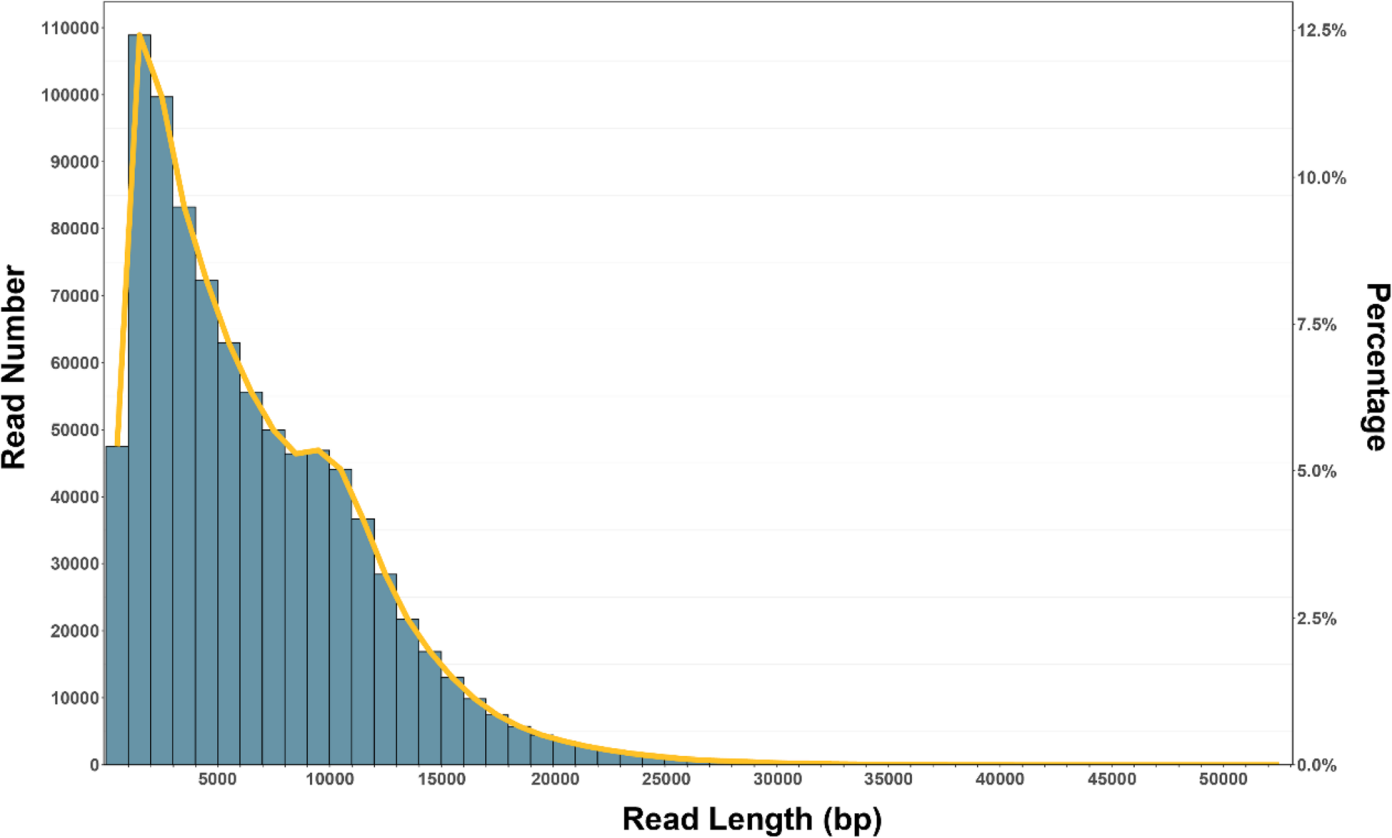
Histogram with raw read length vs. number of raw reads

A total of 5.8 G high-quality subreads were generated with a mean length of 6.6 kb and subreads N50 9.8 kb (Fig. 13). For genome assembly, the subreads were first rectified with canu [86] to obtain trimmed reads, and Wtdbg (https://github.com/ruanjue/wtdbg) was used to perform assembly based on these trimmed reads. The draft assembly contigs were then corrected using Pacbio reads with Quiver [87]. Then, the quivered contigs were further polished with Illumina reads by using Pilon [88]. Finally, the corrected genomic sequence was aligned to the nt database (Nucleotide Sequence Database, ftp://ftp.ncbi.nih.gov/blast/db), and the relevant plant, algae and no-hit sequences were retained. The final assembly genome was 24.23 Mb with contig N50 2 Mb, contig number 31. Length of the longest sequence is 2,973,032 bp, and 12 contigs make up for 90% of the total genome. BUSCO [89] analysis showed that 89.4% of the 303 core genes in the eukaryote dataset were complete (Supplementary Table 1), which indicated that most highly conserved genomes were well packed and that the assembly results were reliable.

The interspersed repetitive sequences were predicted using MISA [90]. Two strategies were adopted to annotate repetitive sequences: RepeatMask based on aligning with a database or RepeatModeler [91] based on constructing a de novo repeat database. For gene structure annotation, the de novo prediction method Augustus [92]; homology alignment prediction, such as with GeneWise [93] and EST/cDNA; and genome alignment prediction approach, including PASA [94], were used separately and integrated through EVM [94]. Transposon PSI [95] alignment was performed to remove genes containing transposable elements.

The predicted protein sequences were searched against KEGG, COG (Cluster of Orthologous Groups), NR (Non-Redundant Protein Database), Trembl and Swissprot to predict gene functions and metabolic information through Blastall [94]. A whole-genome blast was performed against the secondary database InterPro to predict conserved sequences and domains of proteins using InterProScan [96]. For noncoding RNA annotation, both strategies, including alignment with the existing noncoding RNA database Rfam [97] and predictions with tRNAscan-SE [98] or RNAmmer [99], were adopted. The major software program versions and parameter settings has been described in the Supplementary Table 13.

This Whole Genome Shotgun project has been deposited at DDBJ/ENA/GenBank under the accession VXJC00000000. The version described in this paper is version VXJC01000000. Gene annotation file and the gene set files have been provided as supplementary files (Supplementary file 1, 2 and 3). And the PacBio data has been deposited at NCBI under BioProject number PRJNA556117.

### Phylogenetic analysis

We investigated the phylogenetic position of *M.homosphaera* by using 18S rDNA, which is a widely used molecular barcode for taxonomic affiliation in phytoplankton [100]. The analysis involved 18 nucleotide sequences from NCBI (Supplementary Table 14), and all positions containing gaps and missing data were eliminated. There were a total of 1765 positions in the final data set. Maximum likelihood (ML) analysis was conducted based on the Tamura-Nei model [101] in MEGA X [102], with 1000 bootstrap repetitions.

### Drawing tool

OrganellarGenomeDRAW 1.3.1 was used to generate Figs. 3 and 4. and Adobe Illustrator CS6 was used to generate other figures.

## Supplementary information

**Additional file 1: Supplementary Table 1.** BUSCO forecast statistics. **Supplementary Table 2.** SSR classification statistics. **Supplementary Table 3.** List of conserved genes in chloroplast. **Supplementary Table 4.** List of conserved genes in mitochondria. **Supplementary Table 5.** carbon meth genes. **Supplementary Table 6.** Genes involved in nitrogen assimilation up to ammonium. **Supplementary Table 7.** Thiamine biosynthesis genes. **Supplementary Table 8.** Lipid metabolic gene comparison. **Supplementary Table 9.** Starch biosynthesis genes. **Supplementary Table 10.** non-mevalonate pathway (DXP) genes. **Supplementary Table 11.** NUMTs List of Mychonastes sp. and other phytoplankton. **Supplementary Table 12.** NUPTs List of Mychonastes sp. and other phytoplankton. **Supplementary Table 13.** The versions of major software and database. **Supplementary Table 14.** Sources of 18S rDNA sequences. **Supplementary Table 15.** Sources of genomic sequences.

## Data Availability

The datasets generated during the current study are available in the NCBI repository under the accession VXJC00000000. And accession numbers of the reference datasets such as the nucleotide sequences and genomic sequences form NCBI repository had been listed in the Supplementary Table 14 and 15.

## Abbreviations

BUSCOs: Benchmarking Universal Single-Copy Orthologs;
SSR: Simple sequence repeat
CDS: Coding sequence
ORF: Open reading frame
GO: Gene ontology
KEGG: Kyoto Encyclopedia of Genes and Genomes
VDE: Violaxanthin de-epoxidase
ZE: Zeaxanthin epoxidase
SOD: Superoxide dismutase
LHC: Light-harvesting complex
ZIP: Zrt, Irt-like protein
VB_12_: Vitamin B12
TCA: Glycerolipid
ACC: Acetyl-CoA carboxylase
KAR: 3-Oxoacyl-ACP reductase
TAG: Triacylglycerol
PDAT: Phospholipid:diacylglycerol acyltransferase
DGAT: Acyl-CoA:diacylglycerol acyltransferase
MVA: Mevalonate pathway
DXP: Nonmevalonate pathway
KCS: 3-Ketoacyl-CoA synthase
FAD: Fatty acid desaturase
